# A New Grey Relational Analysis Model Based on the Characteristic of Inscribed Core (IC-GRA) and Its Application on Seven-Pilot Carbon Trading Markets of China

**DOI:** 10.3390/ijerph16010099

**Published:** 2018-12-31

**Authors:** Lihong Wang, Kedong Yin, Yun Cao, Xuemei Li

**Affiliations:** 1 School of Economics, Ocean University of China, Qingdao 266100, China; wanglihong@stu.ouc.edu.cn (L.W.); yinkedong@ouc.edu.cn (K.Y.); caoyunouc@126.com (Y.C.); 2 Ocean Development Research Institute, Major Research Base of Humanities and Social Sciences of Ministry of Education, Ocean University of China, Qingdao 266100, China; 3 College of Oceanic and Atmospheric Sciences, Ocean University of China, Qingdao 266100, China

**Keywords:** inscribed core grey correlation, carbon trade prices, grey relational analysis model

## Abstract

In recent years, the study of the factors affecting the carbon trading price plays an important role in promoting the carbon trading markets and the sustainable development of green economy. However, due to the short establishment time of China’s carbon trading market, the carbon trading price data of the pilot markets were not complete and have the typical characteristics of poor information. The traditional grey correlation model cannot effectively identify the volatility and the grey correlation coefficient of trading data. In this paper, an inscribed cored grey relational analysis model (IC-GRA) is constructed by extracting the values of the triangle inscribed center of the time series sample. Through numerical examples and empirical analysis, it is verified that IC-GRA not only satisfies the four axioms of traditional grey correlation but also avoids the influence of outliers of time series fluctuation and improves the discriminability of the grey correlation coefficient. The empirical results of the IC-GRA model in China’s seven pilot carbon trading markets show that: 1. among international carbon trade factor, the biggest influence factor carbon trade price is different in pilot markets. The price of natural gas has a greater correlation with the carbon price of carbon trading markets in Shenzhen, Guangzhou, and Chongqing. The futures price of Certified Emission Reduction (CER) has a strong correlation with the carbon price of Shanghai and Beijing carbon trading markets; the price of Hubei carbon trading market is the largest related to crude oil future price in the New York Mercantile Exchange ( NYMEX). 2. Air Quality Index (AQI) is most relevant to the market carbon price of carbon trading, followed by the trading turnover and trading volume of the carbon trading market. Therefore, studying the carbon trading price of the carbon trading market plays a positive role in improving the sustainable development in those areas.

## 1. Introduction

### 1.1. Introduction of Factors Influencing Carbon Emissions Trading and Carbon Pricing

**Figure 1:**
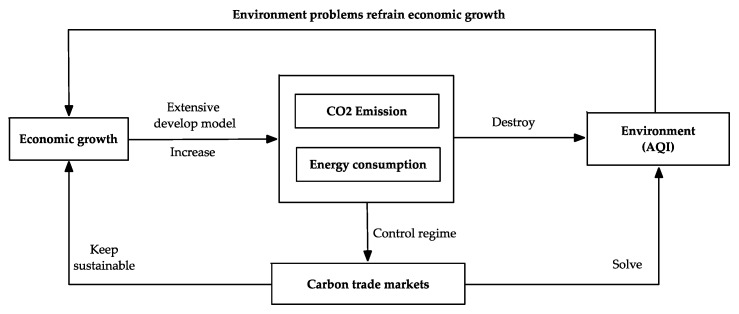
Relationship diagram of economic growth, carbon trade market, and environment.

Our lives and economy have improved immensely with industrialization. However, such developments are at the cost of using vast amounts of fuel energy, increasing carbon dioxide emissions, and endangering the ecosystem and environment. The sustainable development of global economy and society are threatened [1,2]. Carbon emission’s trading is a market mechanism aimed at reducing emissions of greenhouse gas. Adoption of United Nations Framework Convention on Climate Change (UNFCCC) in 1992 and establishment of the Kyoto Protocol promoted implementations of carbon emissions trading system in many countries. Since putting the system into operation in 2005, the European Union now have the largest and most developed carbon trading system in the world, accounting for 90% of global transactions [3]. Emissions Trading System (ETS) or carbon emission reduction program have subsequently been set up in New Zealand, Tokyo, Australia, Canada, and Switzerland. The carbon emissions trading system improves the global energy-environment-economic issues. The relationship between economic growth, carbon trade markets, and environment demonstrates as shown in Figure 1. This paper adopts AQI value to describe the environmental problems. The smaller the index is, the better air quality is. Different scholars hold different views on relationship between carbon trade price and AQI [4,5]. Accompanied by the development of economy, the emission of carbon dioxide will increase. As limitation of carbon quota will increase the demand of carbon trade, the carbon trade price will increase as the result. In this way, the emission of carbon dioxide can be controlled, which will improve the air quality. Therefore, we suppose there is negative relationship between carbon trade price and AQI.

As a member of the Tokyo Protocol, a major consumer of energy and a large carbon emissions country, China has actively taken measures to improve the efficiency of energy utilization and promote clean energy consumption, including passing the Renewable Energy Law and Energy Specific Regulations. In the fifth Plenary Session of the Eighteenth Communist Party of China (CPC) Central Committee, the idea of “green development” was put forward as one of the five important concepts related to the overall development of the country. The session also promoted low-carbon development. The nineteenth CPC National Congress stressed the importance of a rapid ecological and environmental reform, building a new economic system of green, low-carbon and cycle development. On 9 December 2017, the National Development and Reform Commission formally issued the “National Carbon Emissions Trading Market Construction Program (power generation industry)”. This marked the official beginning of China’s carbon emissions trading system. The National Development and Reform Commission previously conducted emissions trading pilot programs in Beijing, Shanghai, Tianjin, Chongqing, Hubei, Guangdong, and Shenzhen, covering key emission industries ranging from petrochemical, chemical, building materials, iron and steel, nonferrous metals to paper, electricity, and aviation. From June 2013 to June 2014, exchanges in all seven cities and regions started to open. Carbon emissions trading were conducted through combinations of carbon quotas and China Certified Emission Reductions (CCER). As of November 2017, the seven exchanges have accumulated more than two billion tons of carbon dioxide equivalents, with a turnover of more than 4.6 billion Yuan, which will make contribution to descending the quantity of the global greenhouse gases.

To ensure the smooth start and docking of local carbon markets, an efficient price regulation mechanism is key. The correct carbon price can not only improve the efficiency of resource allocations but also effectively reflect the cost of emission reduction. Current research on carbon price mainly focus on analyzing factors influencing carbon price, the correlation between carbon price and other energy prices, and the risk of carbon price fluctuations. Zhong et al. [6] studied the effects of carbon prices on China’s energy prices and price fluctuations and found that fluctuations in carbon price can cause China’s energy prices to change but had little effect on overall prices (on commodity prices). Some scholars also studied the linkage effect of different carbon markets, using Dynamic Conditional Correlation Generalized Auto Regressive Conditional Heteroskedasticity (DCC-GARCH) model to conduct empirical research on the dynamic correlation between domestic and foreign carbon quotas prices [7]. Research on the relationship between carbon emission prices and the stock market are mainly conducted using Autoregressive moving average model (ARMA), Auto Regressive Conditional Heteroskedasticity (ARCH), Generalized Auto Regressive Conditional Heteroskedasticity (GARCH), Generalized Error Distribution-Generalized Auto Regressive Conditional Heteroskedasticity (GED-GARCH), and vector auto regressive model (VAR). Koch et al. [8] examined the rule of corporations in carbon emission levels. Qin and Tao [9,10] found a positive relationship between corporate stock returns and carbon trading futures. Regional carbon emissions market can be influenced by politics, trading system, heterogeneous environment, policies, as well as regional factors and Chinese characteristics [11]. As a relatively new market in China, data is not abundant making it difficult to classic econometric models to study carbon prices. A new approach is required to examine the factors influencing carbon prices in China.

### 1.2. Introduction of Grey Relational Degree

Grey relational analysis is an important branch of grey system theory. It is the cornerstone of grey system analysis, modeling, prediction, and decision-making. The analysis is built upon determining the correlation between time series lines or curves of each factor in the system. The closer the line or curve is, the greater the correlation between the factors, and vice versa [12].

Grey relational analysis (GRA) was created by Professor Deng Julong. Many scholars have then followed this train of thought and put forward various grey relational analysis models, including absolute relational model [13], T type relational model [14], B type and C type relational degree [15,16], and slope relational degree [17]. In recent years, many scholars have proposed new models based on this idea or improving previous models. Zhang et al. [18], based on Deng’s model, proposed a GRA-AR relational model, which considers both absolute and relative differences. Xie et al. [19] proposed a grey geometric relational model. Liu et al. [20,21,22,23] based on the integral area of folded lines built a grey absolute relational model and a similarity-perspective grey relational. Shi constructed the grey periodic relational degree and the grey amplitude relational from different perspectives. Zhang et al. [24] used the principle of vector projection, proposed a new grey projection relational model. To investigate the similarity and correlation of dynamic changes between time series, Li et al. [25] proposed a grey rate of change relational degree.

In recent years, the Grey relational theory has matured. It has been widely applied in economics, social science, industry, agriculture, mining, transportation, education, medicine, ecology, water conservancy, geology, and aviation. Luo et al. [26] applied the Grey relational degree theory to investment decision-making and proved that Grey system theory is effective in uncertain information systems. Zheng et al. [27] used B type absolute relational degree to identify liver cancer cells. In view of the complexity of performance assessment and the limitations of existing performance evaluation methods, Zhang et al. [28] established an employee performance evaluation model based on Grey Relational Analysis. Introducing an improved Grey relational analysis method into the risk assessment of supply chains, Chen et al. [29] established an evaluation model. Zhu [30] combined grey relational analysis method and information entropy theory, proposed a grey entropy relation to fitness assignment strategy. It has been used in relation with differential algorithm and genetic algorithm to solve target flow shop scheduling problems.

Nelabhotla et al. [31] applied Taguchi-based grey relational analysis (TGRA) for the optimization of chemical mechanical planarization (CMP) process-parameters of c-plane gallium-nitride (GaN), in potassium-permanganate/alumina (KMnO_4_/Al_2_O_3_) slurry. Wang [32] applied Grey relational analysis to the optimization of mining costs. Jiang et al. [33] constructed a grey relational model of four real estates and their relation with other estates and industries. Kumar et al. [34] used grey relational grade method to compare two different rapid prototyping systems based on dimensional performance. There are many scholars improve the Grey relational analysis and prediction models in many fields [35,36,37,38,39,40,41].

### 1.3. Research Motivation and Content

There are still shortcomings with traditional grey relational models, such as a grey relational model based on area. When a time series changes its position, the overall shape does not change but correlation results have changed. It no longer has the characteristics of an affinity. This grey relational model is based on the area between lines which can only reflect the similarity between series. The lines themselves is a poor representation of series characteristics and difficult to show specific periodic fluctuations. Most of the existing relational models are only suitable for isochronous equidistance sequences, which greatly limits their application. To overcome these shortcomings, this paper proposes a relational model based on center coordinates of an inscribed circle of a triangle. The unique coordinates hold the characteristics of a change in the time series. The degree of correlation between variables can be observed from the range of change and the direction of change.

In addition, examining factors influencing the price of carbon emissions trading provides support for the establishment and improvement of China’s unified carbon trading system. China’s carbon trading emissions market only just opened, acquiring carbon emissions trading data acquisition is not easy. The process has the typical characteristics of “small sample and little information”. This paper first provides a comprehensive literature review of grey relational model and the relationship between China’s carbon emissions futures market prices and their influencing factors. This is followed by an introduction of basic concepts concerning inscribed circle of a triangle. A model using center coordinates of the inscribed circle of a triangle is constructed to overcome shortcomings of traditional grey relational model. Properties of the new model will be discussed. Next, the model is applied to explore the correlation between price and its influencing factors of China’s carbon emissions trading market, verifying the effectiveness, feasibility and superiority of the model. The final section is concluding remarks and prospects.

## 2. Establishment and Properties of the Inscribed Core Grey Relational Analysis Model

### 2.1. Feature Extraction

This article is based on the theory of T relational analysis, which transfers the traditional grey relational into circle arithmetic functions between different segments. It can be defined as the inscribed core grey relational analysis (IC-GRA) model. The main thought of model is to construct a triangle based on data, which decide the only inscribed circle. With respect to the discrete time sequence, the similarity of the change rate is determined by the size of the change rate in each corresponding period of period of $\Delta t_{k}=t_{k}-t_{k-1}$, (k=2,3,⋯,n). The closer the change rate in period $\Delta t_{k}$ is, the greater the correlation coefficient, and vice versa. The use the inscribed center reconstructs a new line, which may weaken the trend and describe a reasonable relationship.

**Figure 2:**
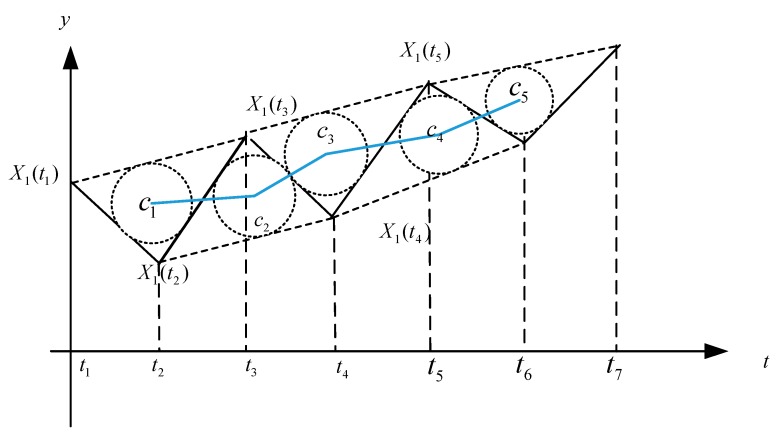
Description of the inscribed core of piecewise linear.

Time series belongs to typical discrete series, which can be described into many descriptions based on recent study. This article is mainly adopting the piecewise linear, which is an important method to extract columns feature and dimensionality reduction. For the time series, as the fixed observation points, it can be regarded as adaptive piecewise liner. Therefore, the piecewise linear can be described as follows:

(1) $$X_{i}=\{(x_{i}(t_{1}),x_{i}(t_{2})),\cdots ,(x_{i}(t_{k-1}),x_{i}(t_{k})),\cdots ,(x_{i}(t_{m-1}),x_{i}(t_{m}))\}$$

where, $t_{k}$ represent the points of time series; i represent the number of this time series, $x_{i}(t_{k})$ represents the value of the *m*-th time series at time $t_{k}$; $x_{i}(t_{k-1}),x_{i}(t_{k})$ represent the start points and the ends points at *k*-th line. Moreover, it need add symbol function avoid completing. The definitions are as follows:

**Definition** **1.** *Based on the piecewise liner of time series, use 2 time periods and the adjacent segments of* $\left(x_{i}(t_{k-1}),x_{i}(t_{k})\right)$ *and* $\left(x_{i}(t_{k}),x_{i}(t_{k+1})\right)$ *which may construct the only triangle, then definite the* $X_{i}=\{r_{i}(t_{2}),r_{i}(t_{3}),\cdots ,r_{i}(t_{k-1})\}$ *as the sequence of the triangle, where k dominate the number of the observation;* $r_{i}(t_{k})$ *represents the triangle constructed by the* $\left(x_{i}(t_{k-1}),x_{i}(t_{k})\right)$ *and* $\left(x_{i}(t_{k}),x_{i}(t_{k+1})\right)$ *at i-th time series as shown in Figure 2. In order to subscribe the time series transfer, IC-GRA model selects the vertical coordinates of the inscribed triangle as the new time series as shown in Figure 2.*

Assume the coordinate of the center of inscribed circle can be described as follows:

(2) $$c_{i}(t_{i})=(a*x_{i}t_{k}+b*x_{i}t_{k+1}+c*x_{i}t_{k+2})/(a+b+c),(a*t_{k}+b*t_{k+1}+c*t_{k+2})/(a+b+c)$$

where,

(3) $$a=\sqrt{{\left[x_{i}t_{k+1}-x_{i}t_{k}\right]}^{2}+{\left(t_{k+1}-t_{k}\right)}^{2}}$$

(4) $$b=\sqrt{{\left[x_{i}(t_{k})-x_{i}(t_{k-1})\right]}^{2}+{\left(t_{k}-t_{k-1}\right)}^{2}}$$

(5) $$c=\sqrt{{\left[x_{i}(t_{k+1})-x_{i}(t_{k-1})\right]}^{2}+{\left(t_{k+1}-t_{k-1}\right)}^{2}}.$$

### 2.2. Relation Analysis

Assume the interval is [p,q], and then set the following formula as the variation function of the corresponding radian series from $t_{k-1}$ to $t_{k}$:

(6) $$y_{i}(t_{k})=c_{i}(t_{k})-c_{i}(t_{k-1})\;k=1,2,\cdots ,n-2.$$

**Definition** **2.** *Calculation of the vector and relation*

*Assume there are two-time series at* [p,q] *, which is* $X_{1}=\{c_{1}(t_{2}),c_{1}(t_{3}),\cdots c_{1}(t_{k-1})\}$ *,* $X_{2}=\{c_{2}(t_{2}),c_{2}(t_{3}),\cdots c_{2}(t_{k-1})\}$ *, respectively. Therefore, define the correlation coefficient could be as follows:*

(7) $$\xi_{0i}(k)=\frac{1}{1+\left|y_{0}(k)-y_{i}(k)\right|}.$$

*In order to effectively distinguish the grey relational coefficient generated by two different time series, two comparative variables have been introduced into the Formula (7), which are the subtraction variable* $\left|\left|y_{0}(t_{k})\right|-\left|y_{i}(t_{k})\right|\right|$ *. When the variation function of two time series in each* $\Delta t_{k}$ *is similar, two comparative variables will tend to be 0, then* $\xi_{0i}(k)$ *will tend to 1, and vice versa.*

**Definition** **3.** *Calculation of the grey relation*

*Assume there are two-time series at* [p,q] *, which is* $X_{1}=\{c_{1}(t_{2}),c_{1}(t_{3}),\cdots c_{1}(t_{k-1})\}$ *,* $X_{2}=\{c_{2}(t_{2}),c_{2}(t_{3}),\cdots c_{2}(t_{k-1})\}$ *, respectively. Moreover, there is non-negative sequence. Therefore:*

(8) $$m=\frac{1}{p-q}\sum_{k=3}^{k-1}\Delta t_{k}\cdot \xi (t_{k}).$$

$X_{1}$ *and* $X_{2}$ *can be regarded as the inscribed core grey relation.*

### 2.3. Property Analysis

**Theorem** **1.** *The correlation of inscribed core model need accord with the properties as follows:*

*(1)* $\left|m_{0i}\right|\leq 1$ *(Correlation degree)*

*(2) Uniqueness, which means independence of disturbances.*

*(3) Proximity. The smaller* $\left|c_{0}(t)-c_{i}(t_{k})\right|$ *is, the bigger* $m(c_{0}(t_{k}),c_{i}(t_{k}))$ *is. Where* $m(X_{0},X_{i})$ *is the grey relational degree,* $m(c_{0}(t_{k}),c_{i}(t_{k}))$ *is the correlation coefficient.*

*(4) Symmetry.*$m_{0i}=m_{i0}$.

*(5) Rank preservation. As it still uses the coordinate values of the data, which demonstrate the length of the Cross-ordinate, it avoids the influence of dimension on the computational results. Therefore, the inscribed core model still keeps the rank preservation.*

**Proof.**  (1) We can obtain from definition1, so:

$$-1<y_{m}(t_{k})<1\Leftrightarrow -1\leq m_{0i}(t_{k})\leq 1,i=1,2,\cdots ,m;k=2,3,\cdots ,n,[p,q]=\overset{n-2}{\underset{k=1}{\cup }}\Delta t_{k}\;\Leftrightarrow -1\leq m_{0i}=\frac{1}{q-p}\sum_{k=2}^{n}\Delta t_{k}\cdot \xi_{0i}(t_{k})\leq 1$$

(2) According to the definitions of the grey relational coefficient and the grey relational degree of the IC-GRA model, property (2) is obvious. That value determines the sequence I uniquely determined, and from the system affect other sequences.

(3) The grey relational coefficient and the grey relational degree of the IC-GRA model are only connected with the two sequences involved. Once the sequences are determined, the radius are determined. Therefore, $m_{0i}$ of certain sequences are confirmed and are not affected by other sequences in the system.

(4) This property is correct due to property (3). □

**Theorem** **2.** *The property of parallel.*

*Assume there are two time series,*$X_{0}=\{x_{0}(t_{1}),x_{0}(t_{2}),\cdots ,x_{0}(t_{n})\}$*and*$X_{i}=\{x_{i}(t_{1}),x_{i}(t_{2}),\cdots ,x_{i}(t_{n})\}$ (i=1,2,⋯,m)*, and their grey relational degree is* $\xi_{0i}$*, which satisfies* $\left|\xi_{0i}\right|\leq 1$ *and* $\xi_{0i}=1$*, if and only if , (*k=1,2,⋯,n*, c is a constant), then* $X_{0}$ *and* $X_{i}$ *are parallel, that is, the IC-GRA model satisfies the normativity of GRA.*

**Proof.**  As

$$\left|\xi_{0i}\right|\leq 1\;\mathrm{and}\;\xi_{0i}=1\Leftrightarrow \xi_{0i}(t_{k})=1\Leftrightarrow y_{0}(t_{k})=y_{i}(t_{k})\Leftrightarrow c_{0}(t_{k})-c_{0}(t_{k-1})\;=c_{i}(t_{k})-c_{i}(t_{k-1})\Leftrightarrow c_{0}(t_{k})-c_{0}(t_{1})=c_{i}(t_{k})-c_{i}(t_{1})\Leftrightarrow c_{i}(t_{k})=\;c_{0}(t_{k})-c_{0}(t_{1})+c_{i}(t_{1})\Leftrightarrow c_{i}(t_{k})=c_{0}(t_{k})+g\Leftrightarrow X_{i}(t_{k})=X_{0}(t_{k})+g\Leftrightarrow X_{0}\;\mathrm{and}\;X_{i}\;\mathrm{are}\;\mathrm{parallel}.$$

And $g=c_{i}(t_{1})-c_{0}(t_{1})$. That is, the model of IC-GRA satisfies the characteristic of GRA’s normativity. □

**Figure 3:**
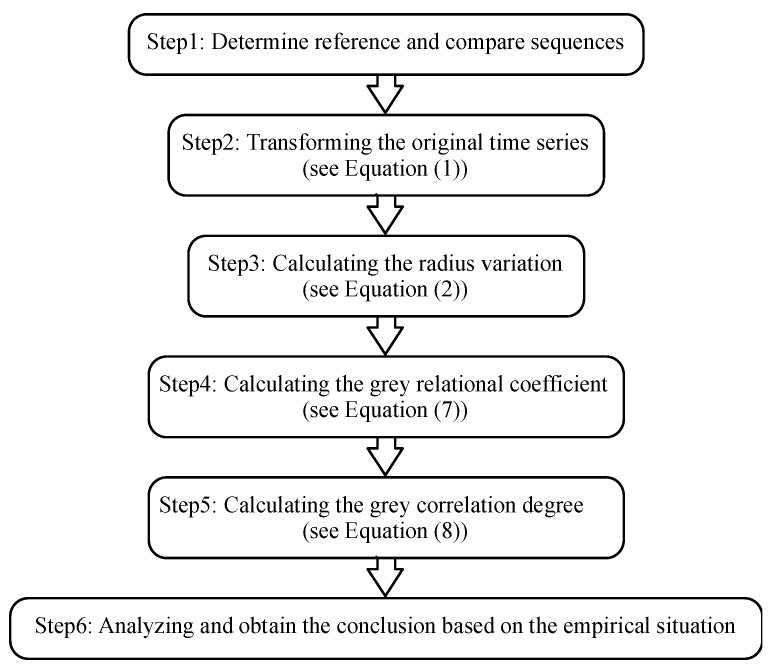
Steps for inscribed cored grey relational analysis model (IC-GRA) model calculations.

The Steps to Calculating the IC-GRA model demonstrated in Figure 3:

### 2.4. Numerical Example

For the following four sequences:

$$X_{0}=\left\{1,2,2.5,2.5,3,5,6,5.4,6.3,6.9,7.5,6,8.2,9.3,9.8\right\}\;X_{1}=\{1,1.8,2.3,2.4,2.8,4.8,5.8,5.2,5.9,6.4,6.7,7.3,6.9,7.6,8.9\}\;X_{2}=\{1,1.8,2.3,2,3,4.1,5.7,5.3,5.5,6.6,6.5,7.4,6.8,7.8,8.8\}\;X_{3}=\left\{1,2.01,2.5,2.4,2.7,4.5,5.8,5.1,5.85,6.3,6.6,7.2,6.8,7.58,8.9\right\}$$

where, $X_{0}$ is the reference sequence, $X_{1}$, $X_{2}$, and $X_{3}$ are comparison sequences. This paper uses IC-GRA model to calculate the correlation degree to compares with Deng’s correlation degree, Grey absolute correlation degree, and Grey slope correlation degree. The calculation results are shown in the following Table 1.

According to the Table 1, we can see that IC-GRA model has the same grey correlation order with Grey absolute correlation degree and Grey slope correlation degree. The correlation degree of the IC-GRA model has been greatly improved over the traditional Deng’s correlation degree. We can conclude that the grey correlation order is accurate and that IC-GRA model can accurately represent the grey correlation degree of different sequences. Next, we discuss the discriminability of the grey relational coefficient.

We use variance to measure the discriminability of the grey relational coefficient. The larger the variance of the grey relational coefficient, the more discrete it is, and we can say the grey relational coefficient has greater discriminability. Owing to the small variance of four methods, we employ normalized methods to improve the discrimination degree of grey relational coefficient. According to the Table 2, IC-GRA model has the largest variance and highest discrimination degree value (DD-value). It proves that the IC-GRA model has a greater degree of discriminability and it can more clearly reflect the true development trend of different sequences, which promotes the superiority and practicability of the IC-GRA method.

## 3. Empirical Analysis

### 3.1. Variables and Data Source

All pilot cities and regions have formulated market access rules accordingly. Most pilot cities and regions have included major companies from power (six cities), iron and steel (five cities), chemical (four cities), cement, petrochemical and paper industries in their carbon trading. Shenzhen and Tianjin carbon market also included companies from construction, transportation industries as sources of emission. According to a low-carbon action list of enterprises and energy saving target, a total of 2274 companies is distributed across the seven cities. The basic information about the carbon markets and the rules for administration are shown in Table 3. All cities except Hubei province use the annual emission of carbon dioxide as a criterion in determining the market access. Hubei province uses energy consumption, a criterion also used in Chongqing and Guangdong carbon trade market. In line with domestic and foreign research on the subject and availability of data, this paper choses carbon trade price of the seven cities as the reference series. The influence factors are chosen from the three aspects. The first aspect is the international carbon trade factor including CER futures price, EUA futures price. The second aspect is the energy price which include WTI index, NYMEX crude oil future price, Chinese coal price, Chinese gasoline price, and natural gas price. The third aspect is the economy development, including the CSI300 and Shanghai industrial index. Data from July 2014 to January 2018 have been weekly selected for consistency. All data can be found on the official carbon trading website, WIND database and the Shanghai Stock Exchange website.

**Figure 4:**
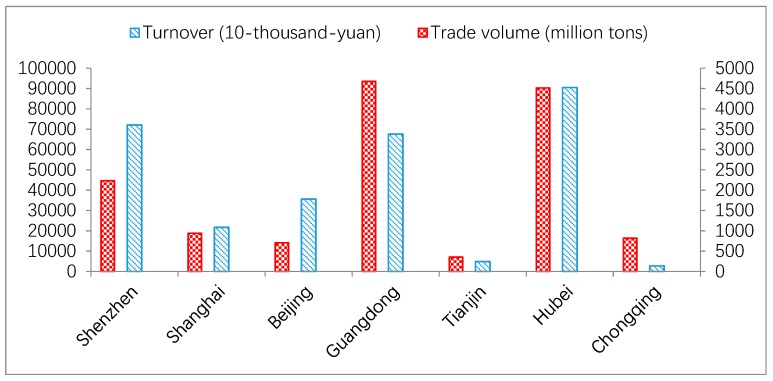
Seven-pilot carbon trade market’s cumulative trade volume and turnover from May 2014 to January 2018.

**Figure 5:**
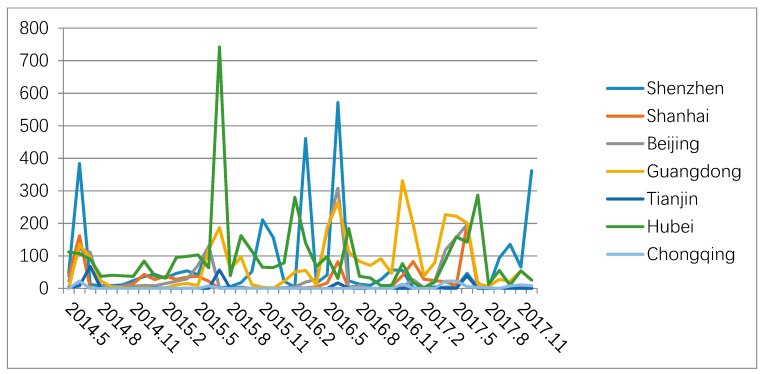
Monthly trade turnover in seven-pilot carbon trade markets.

**Figure 6:**
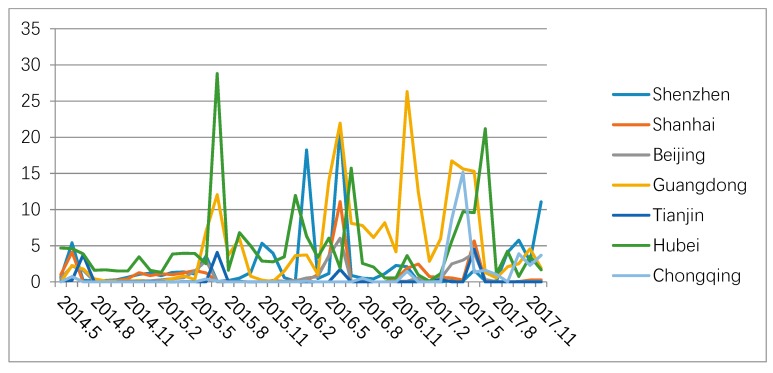
Monthly trade volume in seven-pilot carbon trade markets.

As can be seen from Figure 4, Shenzhen, Shanghai, and Beijing carbon trade markets display high trading price with low trading volume, while Guangdong, Tianjin, and Chongqing carbon trade market show low trading price with high trading volume. Based on Figure 5 and Figure 6 above, it is obviously to find the carbon trade price and trading volume in those pilot regions fluctuated wildly.

### 3.2. Correlation Degree of the Factors in the Carbon Market

**Figure 7:**
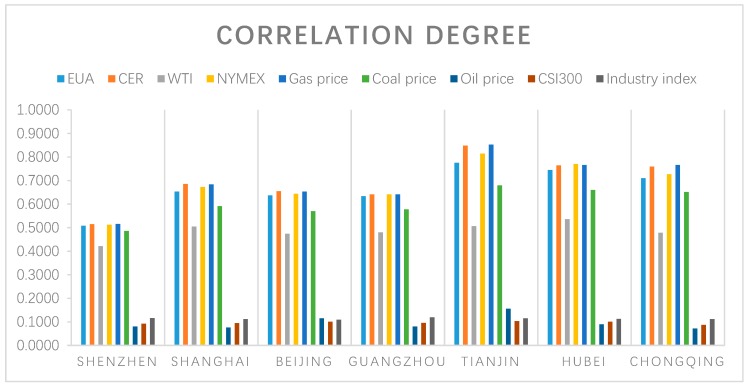
Correlation degree of the influence factors.

By using the Equation (2), it can get the coordination of inscribed core in different factors. According to the Equation (6), it can estimate the value of correlation coefficient. Based on the Equation (7), it can get the correlation degree m as follows:

Based on the Table 4 and Figure 4 above, the estimated results demonstrate that although there are different results in different carbon markets, it still remains some common influence factors. There is a larger correlation degree with the carbon trade price of the Shenzhen, Guangzhou, and Chongqing market. It is also found the futures price of CER has a significant correlation degree in Shanghai and Beijing market. The biggest correlation degree factors in Hubei carbon market is the NYMEX. As can be seen from Figure 7, it is apparent to found out the oil price, CSI300 and the Shanghai Industrial index have a minor correlation degree in those seven-pilot carbon trade markets. The reason for this phenomenon is the amounts of the controlled listed companies are very few.

### 3.3. Comparative Analysis with Traditional Grey Relational Methods

We select the traditional Deng’s grey relational model, grey slope relational model and grey absolute relational model to compare the grey relational degree.

According to the results from Table 5, Table 6 and Table 7, we can calculate the rank of the selected influence factors listed as follows:

Results varied depending on those relational models which can be seen in the above Table 8, Table 9, Table 10 and Table 11. Deng’s relational model considers the proximity of time series and results show that the energy price factors have a relatively high degree of proximity, inferring strong correlation, followed by grey slope relational model examines the problem from a developmental trend similarity perspective. High-speed economic development is no longer pursued by our country and is sustainable development is a long-term plan. In addition, with the exception of Hubei province, the correlation between the prices of pilot carbon rights and energy prices is still high. The correlation between the price of each pilot carbon right and the economic situation is highly close to the correlation with the industrial conditions, indicating that there is still a close relationship between China’s economic development and industrial output. The pilot carbon price and the futures price of the international market products CER all showed a correlation of about 0.582, indicating that the internationalization of China’s carbon pilot projects has achieved success.

Moreover, this paper also estimates the relationship between environment problems and carbon trade market by choosing AQI as independent variable and carbon price, trade volume and turnover as dependent variables. We adopt the IC-GRA model to calculate their correlational degree. The rank of relational degree is shown in Table 12. No matter in which carbon trade market, the biggest influence factor is carbon trade price. The following influence factors are trade turnover and volume, respectively. The empirical results also show there is a negative relationship between AQI and carbon trade price in Shenzhen and Shanghai market which is the same as we discuss in introduction part, while there is a positive relationship in the rest markets. Negative relationship demonstrates the national green development policy effectively enforced and the carbon trade markets are regulated in Shenzhen and Shanghai. The positive relationship performs that there is a difficulty to carry out the whole national policies or plans and the carbon trade market still imperfect.

## 4. Conclusions

Based on the study of the traditional grey correlation, this paper changes the piecewise linear of time series into inscribe core type, from which to get further the calculation method of grey correlation coefficient and correlation degree. In this study, the model ensures the symmetry of grey relational model, uniqueness, preserving order and other characteristics, thus ensuring the completeness and accuracy of the calculation results. Moreover, as it extracts the basic feature of the fluctuated time series, which may provide a reasonable predict of the tendency of the difference and change of the time-series. The relational sequence order of the IC-GRA model is similar with the traditional models. As the model enlarges the correlation coefficient, it provides a higher variance which can improve the disperse degree of the results and easy to distinguish differences. In the future, the IC-GRA model can adopt the circumcircle and orthocenter of the triangle to estimate the grey relational degree and similarity. Moreover, it also can be applied on the poor information and volatility data.

The applications on analyses influence factors for the carbon trade price in seven-pilot carbon trade markets illustrate the effectiveness and practicality of the IC-GRA model. From the empirical analysis, it shows the relational degree of each factor that influences the carbon price. The seven-pilot carbon trade markets present different results—the international influence factors have larger correlational degree and follows by the energy price. The last factor is the economic index. Understanding the rank of the factor will guide the development and the construct of the national carbon trade market and promoting international cooperation. Moreover, AQI, which reflects the environment quality, has a closer correlational degree with the carbon trade price compared with the trade turnover and trade volume in pilot areas. The trading and activity of carbon trade market will restrain the emission of carbon dioxide and help to improve air quality. Therefore, encouraging enterprises and government to actively participate and consummate the regulation of the carbon trade market will help the environment issues.

## Figures and Tables

**Table 1:** Comparison of the several grey correlation degrees.

m	$m_{01}$	$m_{02}$	$m_{03}$	The Grey Correlation Order
IC-GRA model	0.9459	0.8183	0.8869	$m_{01}>m_{03}>m_{02}$
Deng’s correlation degree	0.5485	0.5554	0.5482	$m_{02}>m_{01}>m_{03}$
Grey absolute correlation degree	0.9405	0.9316	0.9362	$m_{01}>m_{03}>m_{02}$
Grey slope correlation degree	0.9721	0.9310	0.9715	$m_{01}>m_{03}>m_{02}$

**Table 2:** The discriminability analysis of the grey relational coefficient.

The Difference of the Grey Relational Coefficient	Variance	DD-Value
IC-GRA model	0.004076081	0.873214
Deng’s correlation degree	0.000016595	0.003555
Grey absolute correlation degree	0.000019508	0.004179
Grey slope correlation degree	0.000555723	0.119052

**Table 3:** Basic information about the carbon markets and the admission rules.

Carbon Markets	Shenzhen	Shanghai	Beijing	Guangdong	Tianjin	Hubei	Chongqing
Start Time	June 2013	November 2013	November 2013	December 2013	December 2013	December 2014	July 2014
Amount of initial controlled enterprises	635	197	490	211	114	138	242
Controlled enterprise’s standard	The average emission amount exceeds 10,000 tons from 2009 to 2011.	The enterprise’s emission amount over 20,000 tons from 2009	The enterprise’s emission amount over 10,000 tons.	The enterprise’s emission amount over 20,000 tons.	The enterprise’s emission amount over 20,000 tons from 2011 to2014	The enterprise’s coal conversion over 60,000 tons.	The enterprise’s coal conversion over 10,000 tons from 2013 to 2015.
Allocation Methods	Historical emission method and datum line methods	Historical emission method and datum line methods	Historical emission method	Historical emission method and datum line methods	/	/	Datum line methods

**Table 4:** Correlation degree.

m	EUA	CER	WTI	NYMEX	Gas Price	Coal Price	Oil Price	CSI300	Industry Index
Shenzhen	0.5082	0.5149	0.4211	0.5124	0.5156	0.4857	0.0803	0.0924	0.1156
Shanghai	0.6530	0.6851	0.5042	0.6722	0.6838	0.5909	0.0762	0.0948	0.1115
Beijing	0.6371	0.6544	0.4739	0.6437	0.6533	0.5697	0.1150	0.1001	0.1093
Guangzhou	0.6333	0.6410	0.4800	0.6408	0.6414	0.5771	0.0804	0.0950	0.1191
Tianjin	0.7749	0.8481	0.5063	0.8143	0.8521	0.6793	0.1553	0.1027	0.1150
Hubei	0.7447	0.7645	0.5355	0.7705	0.7661	0.6599	0.0898	0.1000	0.1128
Chongqing	0.7096	0.7595	0.4784	0.7268	0.7657	0.6510	0.0716	0.0871	0.1114

**Table 5:** Estimated degree of correlation in Deng’s grey relational model.

	EUA	CER	WTI	NYMEX	Gas Price	Coal Price	Oil Price	CSI300	Industry Index
Shenzhen	0.8092	0.6076	0.9590	0.9410	0.8637	0.8702	0.9317	0.7304	0.7438
Shanghai	0.8411	0.6290	0.9370	0.9377	0.9064	0.8994	0.9320	0.7585	0.7733
Beijing	0.9013	0.6392	0.8786	0.9086	0.9663	0.9629	0.9317	0.8025	0.8210
Guangzhou	0.7732	0.6036	0.9236	0.8956	0.8202	0.8252	0.8784	0.7054	0.7171
Tianjin	0.8093	0.5973	0.9396	0.9266	0.8685	0.8735	0.9256	0.7290	0.7424
Hubei	0.8827	0.6079	0.9022	0.9259	0.9452	0.9221	0.9408	0.7838	0.7998
Chongqing	0.7902	0.6107	0.8990	0.8901	0.8336	0.8330	0.8819	0.6903	0.7061

**Table 6:** Estimated degree of correlation in grey absolute relational model.

	EUA	CER	WTI	NYMEX	Gas Price	Coal Price	Oil Price	CSI300	Industry Index
Shenzhen	0.5150	0.0345	0.8977	0.5841	0.0069	0.2407	0.6565	0.5188	0.7823
Shanghai	0.5345	0.0763	0.7508	0.7747	0.0394	0.5498	0.6716	0.5149	0.7783
Beijing	0.6345	0.2504	0.6864	0.9529	0.1339	0.6578	0.6731	0.5127	0.7760
Guangzhou	0.5115	0.0266	0.9956	0.5153	0.0053	0.1844	0.6575	0.6745	0.7844
Tianjin	0.5318	0.0706	0.7592	0.7580	0.0342	0.5059	0.6743	0.5152	0.7785
Hubei	0.6385	0.2563	0.6858	0.9551	0.0623	0.6521	0.6536	0.5127	0.7760
Chongqing	0.5359	0.0791	0.7473	0.7820	0.0164	0.5710	0.6546	0.5148	0.7782

**Table 7:** Estimated degree of correlation in grey slope relational model.

	EUA	CER	WTI	NYMEX	Gas Price	Coal Price	Oil Price	CSI300	Industry Index
Shenzhen	0.8838	0.8688	0.8866	0.8831	0.8939	0.8941	0.8924	0.8895	0.8884
Shanghai	0.9260	0.9135	0.9298	0.9213	0.9499	0.9486	0.9433	0.9393	0.9382
Beijing	0.9422	0.9247	0.9467	0.9347	0.9647	0.9631	0.9537	0.9516	0.9508
Guangzhou	0.9094	0.8946	0.9101	0.9017	0.9238	0.9232	0.9200	0.9181	0.9162
Tianjin	0.9439	0.9347	0.9471	0.9356	0.9781	0.9748	0.9636	0.9606	0.9588
Hubei	0.9439	0.9268	0.9484	0.9434	0.9669	0.9665	0.9613	0.9602	0.9579
Chongqing	0.8913	0.8733	0.8893	0.8820	0.9141	0.9119	0.9051	0.9020	0.9003

**Table 8:** Rank of the selected influence factors in IC-GRA model.

	EUA	CER	WTI	NYMEX	Gas Price	Coal Price	Oil Price	CSI300	Industry Index
Shenzhen	4	2	6	3	1	5	9	8	7
Shanghai	4	1	6	3	2	5	9	8	7
Beijing	4	1	6	3	2	5	7	9	8
Guangzhou	4	2	6	3	1	5	9	8	7
Tianjin	4	2	6	3	1	5	7	9	8
Hubei	4	3	6	1	2	5	9	8	7
Chongqing	4	2	6	3	1	5	9	8	7

**Table 9:** Rank of the selected influence factors in Deng’s grey relational model.

	EUA	CER	WTI	NYMEX	Gas Price	Coal Price	Oil Price	CSI300	Industry Index
Shenzhen	6	9	1	2	5	4	3	8	7
Shanghai	6	9	2	1	4	5	3	8	7
Beijing	5	9	6	4	1	2	3	8	7
Guangzhou	6	9	1	2	5	4	3	8	7
Tianjin	6	9	1	2	5	4	3	8	7
Hubei	6	9	5	3	1	4	2	8	7
Chongqing	6	9	1	2	4	5	3	8	7

**Table 10:** Rank of the selected influence factors in grey slope relational model.

	EUA	CER	WTI	NYMEX	Gas Price	Coal Price	Oil Price	CSI300	Industry Index
Shenzhen	7	9	6	8	2	1	3	4	5
Shanghai	7	9	6	8	1	2	3	4	5
Beijing	7	9	6	8	1	2	3	4	5
Guangzhou	7	9	6	8	1	2	3	4	5
Tianjin	7	9	6	8	1	2	3	4	5
Hubei	7	9	6	8	1	2	3	4	5
Chongqing	6	9	7	8	1	2	3	4	5

**Table 11:** Rank of the selected influence factors in grey absolute relational model.

	EUA	CER	WTI	NYMEX	Gas Price	Coal Price	Oil Price	CSI300	Industry Index
Shenzhen	6	8	1	4	9	7	3	5	2
Shanghai	6	8	3	2	9	5	4	7	1
Beijing	6	8	3	1	9	5	4	7	2
Guangzhou	6	8	1	5	9	7	4	3	2
Tianjin	5	8	2	3	9	7	4	6	1
Hubei	6	8	3	1	9	5	4	7	2
Chongqing	6	8	3	1	9	5	4	7	2

**Table 12:** Rank of relational degree of AQI (Air Quality Index) and price, trade volume, and turnover selected influence factors in IC-GRA model.

AQI	Shenzhen	Shanghai	Beijing	Guangzhou	Tianjin	Hubei	Chongqing
Price	1	1	1	1	1	1	1
Trade volume	3	3	3	3	3	3	3
Turnover	2	2	2	2	2	2	2
